# Supplementary material for: Ethnobotanical Survey of Local Flora Used for Medicinal Purposes among Indigenous People in Five Areas in Lagos State, Nigeria
Source: Plants (Basel). 2022 Feb 25;11(5):633. doi: 10.3390/plants11050633 (PMC8912796; doi:10.3390/plants11050633)
Supplement: Supplementary file 1 [file plants-11-00633-s001.zip › plants-1574822-supplementary.pdf]

Table S1. Inventory, taxonomical profiles and life-form for plants used for managing diverse health conditions/diseases in five (5) selected locations in Lagos State of Nigeria.

S/N	Botanical name	Family	Local name (Yoruba)	Voucher number	Part(s) used	Life-form
1	*Abrus precatorius* L.	Fabaceae	Ominsinmisin	103251	Leaf, Stem	Climbers
2	*Acalypha fimbriata* Schumach. & Thonn.	Euphorbiaceae	Jinwini	103252	Leaf, Stem	Herb
3	*Acanthospermum hispidum* DC.	Asteraceae	Dagunro	103253	Leaf, Root	Herb
4	*Achyranthes aspera* L.	Amaranthaceae	Areyinkosun Aboro	103254	Leaf, Stem	Herb
5	*Adenia lobata* (Jacq.) Engl.	Passifloraceae	Kurere	103255	Stem	Climbing shrub
6	*Ageratum conyzoides* (L.) L.	Asteraceae	Imi esu, Rerinkomi	103256	Leaf	Herb
7	*Albizia ferruginea* (Guill. & Perr.) Benth.	Fabaceae	Ayunre	103257	Stem, Root	Tree
8	*Albizia lebbeck* (L.) Benth.	Fabaceae	Ayunre, Igbabgo	103258	Root	Tree
9	*Albizia zygia* (DC.) J.F.Macbr.	Fabaceae	Ayunre were	103259	Leaf	Tree
10	*Alchornea cordifolia* (Schumach. & Thonn.) Müll.Arg.	Euphorbiaceae	Ipa, ewe ifa	103260	Leaf, Stem, Root	Shrub/Small tree
11	*Alchornea laxiflora* (Benth.) Pax & K.Hoffm.	Euphorbiaceae	Ijan	103261	Root	Shrub/Small tree
12	*Alternanthera sessilis* (L.) R.Br. ex DC.	Amaranthaceae	Rekureku	103262	Leaf, Root	Herb
13	*Amaranthus spinosus* L.	Amaranthaceae	Tete elegun, teteegun	103263	Stem	Herb
14	*Amaranthus viridis* L.	Amaranthaceae	Tete	103264	Root	Herb
15	*Anacardium occidentale* L.	Anacardiaceae	Kaju	103265	Leaf	Tree

16	*Anchomanes difformis* (Blume) Engl.	Araceae	Ogirisako	103266	Stem	Herb
17	*Annona senegalensis* Pers.	Annonaceae	Abo	103267	Leaf	Shrub/Small tree
18	*Anthocleista djalonensis* A.Chev.	Gentianaceae	Sapo	103268	Stem	Tree
19	*Anthocleista vogelii* Planch.	Gentianaceae	Sapo	103269	Leaf	Tree
20	*Artocarpus communis* J.R.Forst. & G.Forst. (Synonym: *Artocarpus altilis* (Parkinson ex F.A.Zorn) Fosberg)	Moraceae	Jaloke	103270	Leaf	Tree
21	*Aspilia africana* (Pers.) C.D.Adams	Asteraceae	Yunyun	103271	Leaf, Stem, Root	Herb
22	*Asystasia gangetica* (L.) T.Anderson	Acanthaceae	Lobiri	103272	Leaf, Stem	Creeping herb
23	*Azadirachta indica* A.Juss.	Meliaceae	Eke-oyibo, Dogoyaro	103273	Stem	Tree
24	*Baphia nitida* Lodd.	Fabaceae	Irosun	103274	Leaf	Shrub
25	*Barleria opaca* (Vahl) Nees	Acanthaceae	Apado, Arenikosun	103275	Root	Shrub
26	*Bidens pilosa* L.	Asteraceae	Abere-oloko	103276	Root	Herb
27	*Boerhavia diffusa* L.	Nyctaginaceae	Etiponola	103277	Stem	Herb
28	*Borreria scabra* (Schumach. & Thonn.) K.Schum. (Synonym: *Spermacoce ruelliae* DC.)	Rubiaceae	Awede	103278	Stem, Root	Herb
29	*Borreria verticillata* (L.) G.Mey. (Synonym: *Spermacoce verticillata* L.)	Rubiaceae	Irawo-ile, Otete, Awede	103279	Leaf	Herb
30	*Bridelia ferruginea* Benth.	Euphorbiaceae	Ira	103280	Stem, Root	Shrub

31	*Bryophyllum pinnatum* (Lam.) Oken	Crassulaceae	Abamoda	103281	Stem, Root	Herb
32	*Caladium bicolor* (Aiton) Vent.	Araceae	Eje-jesu	103282	Stem, Root	Herb
33	*Calophyllum inophyllum* L.	Calophyllaceae		103283	Leaf	Tree
34	*Calopogonium mucunoides* Desv.	Fabaceae	Werepe ibile	103284	Leaf, Root	Creeping herb
35	*Calotropis procera* (Aiton) Dryand.	Apocynaceae	Bomu-bomu	103285	Leaf	Shrub
36	*Canna indica* L.	Cannaceae	Loborikan, Ido	103286	Leaf, Stem	Herb
37	*Canavalia ensiformis* (L.) DC.	Fabaceae	Ponpondo	103287	Leaf	Climber
38	*Carpolobia lutea* G.Don	Polygalaceae	Osunsun	103288	Root	Shrub/small tree
39	*Celosia argentea* L.	Amaranthaceae	Soko	103289	Leaf	Herb
40	*Centrosema pubescens* Benth.	Fabaceae	Ewa-ahun	103290	Leaf, Root	Climbing herbaceous vine
41	*Chassalia kolly* (Schumach.) Hepper	Rubiaceae	Isepe agbe	103291	Leaf	Shrub
42	*Chromolaena odorata* (L.) R.M.King & H.Rob.	Asteraceae	Akintola	103292	Leaf, Stem, Root	Shrub
43	*Cissampelos owariensis* P.Beauv. ex DC.	Menispermaceae	Jenjoko	103293	Leaf	Climber
44	*Citrus aurantiifolia* (Christm.) Swingle	Rutaceae	Gaingain	103294	Leaf	Tree
45	*Cleistopholis patens* (Benth.) Engl. & Diels	Annonaceae	Apako, Orila	103295	Stem	Tree
46	*Cleome fruticosa* L. (Synonym: *Cadaba fruticosa* (L.) Druce)	Capparaceae	Ekuya	103296	Leaf, Root	Shrub

47	*Clerodendrum capitatum* (Willd.) Schumach & Thonn.	Lamiaceae	Agbosa, Feregede	103297	Leaf, Root	Shrub
48	*Clerodendrum paniculatum* L.	Lamiaceae	Isedun	103298	Stem	Shrub
49	*Clerodendrum umbellatum* Poir.	Lamiaceae	Isedun	103299	Leaf	Shrub
50	*Clerodendrum volubile* P.Beauv.	Lamiaceae	Marugbosanyan, Marugbo	103300	Leaf	Climbing shrub
51	*Cnestis ferruginea* Vahl ex DC.	Connaraceae	Omu aja, Gboyín-Gboyín	103301	Leaf, Stem	Climbing shrub
52	*Cola millenii* K.Schum.	Malvaceae	Obi-edun	103302	Root	Shrub/Tree
53	*Colocasia esculenta* (L.) Schott	Araceae	Isu- koko	103251	Leaf	Herb
54	*Commelina africana* L.	Commelinaceae	Gbagodo, Aikujegure	103303	Leaf	Climber
55	*Commelina erecta* L.	Commelinaceae	Ìlẹ̀kẹ̀ ọ̀pọlọ́	103304	Stem	Herb
56	*Costus afer* Ker Gawl.	Costaceae	Ireke Omode, Tete-eegungun	103305	Root, Stem	Rhizomatous herb
57	*Croton lobatus* L. (Synonym: *Astraea lobata* (L.) Klotzsch).	Euphorbiaceae	Eru	103306	Root	Forb/Herb
58	*Croton zambesicus* Mull.Arg. (Synonym: *Croton gratissimus* Burch.)	Euphorbiaceae	Aje-obale, Aje-kofole	103307	Leaf, Root	Shrub
59	*Cucumeropsis mannii* Naudin	Cucurbitaceae	Egusi Itoo	103308	Root	Creeper
60	*Cyathula prostrata* (L.) Blume	Amaranthaceae	*Sawerepepe*	103309	Leaf	Herb
61	*Cymbopogon citratus* (DC.) Stapf	Poaceae	Ewe tea, Waapa	103310	Leaf, Root	Herb
62	*Cyperus haspans* L.	Cyperaceae	Ayaa-ayaa (Hausa)	103311	Stem	Sedge

63	*Dalbergia saxatilis* Hook.f.	Fabaceae	Ogundu, Ojiji	103312	Root	Climbing shrub
64	*Datura metel* L.	Solanaceae	Apikan, Ewe alujannu	103313	Leaf	Shrub
65	*Desmodium velutinum* (Willd.) DC.	Fabaceae	Ewe oku	103314	Leaf	Shrub
66	*Dichrostachys cinerea* (L.) Wight & Arn.	Fabaceae	Kara, Ajagboluti	103315	Leaf	Tree
67	*Dissotis rotundifolia* (Sm.) Triana (Synonym *Heterotis rotundifolia* (Sm.) Jacq.-Fél.)	Melastomataceae	Awede, Ajagunmorasin, Ogede apara	103316	Leaf	Shrub
68	*Eclipta prostrata* (L.) L.	Asteraceae	Arojoku	103317	Leaf, Root	Herb
69	*Elaeis guineensis* Jacq.	Arecaceae	Ope	103318	Stem, Root	Tree
70	*Eleusine indica* (L.) Gaertn.	Poaceae	Gbegi	103319	Stem, Root	Herb
71	*Eleutheranthera ruderalis* (Swartz) Sch.-Bip.	Asteraceae	Aworo-ona	103320	Leaf	Herb
72	*Emilia coccinea* (Sims) G.Don	Asteraceae	Odundun owo	103321	Leaf, Stem, Root	Herb
73	*Entandrophragma angolense* (Welw.) C.DC.	Meliaceae	Jebo	103322	Stem-bark, Leaf	Tree
74	*Eragrostis namaquennsis* Nees ex Schrad. (Synonym: *Eragrostis japonica* (Thunb.) Trin.)	Poaceae	Agbado esin	103323	Stem	Herb
75	*Erigeron floribundus* (Kunth) Sch.Bip.	Asteraceae	Bị́bị́ị́mbẹ́lẹ́mọ́	103324	Leaf	Herb
76	*Erythrina senegalensis* DC.	Fabaceae	Ologbosere	103325	Root	Shrub/Tree
77	*Euphorbia glaucophylla* Poir. (Synonym: *Euphorbia trinervia* Schumach. & Thonn.)	Euphorbiaceae	Emile	103326	Leaf, Stem	Herb
78	*Euphorbia heterophylla* L.	Euphorbiaceae	Egele	103327	Stem	Herb

79	*Ficus benjamina* L.	Moraceae	Odan	103328	Leaf, Stem-bark	Tree
80	*Ficus capensis* Thunb. (Synonym: *Ficus sur* Forssk.)	Moraceae	Opoto	103329	Stem, Leaf	Tree
81	*Ficus exasperata* Vahl	Moraceae	Epin	103330	Leaf	Shrub
82	*Ficus polita* Vahl	Moraceae	Odan	103331	Stem	Shrub/ small tree
83	*Laportea aestuans* (L.) Chew	Urticaceae	Araailomi	103332	Whole plant	Herb
84	*Gliricidia sepium* (Jacq.) Walp.	Fabaceae	Agunmaniye	103333	Leaf, Stem	Small tree
85	*Glyphaea brevis* (Spreng.) Monach.	Malvaceae	Atori	103334	Leaf	Tree
86	*Gomphrena celosioides* Mart.	Amaranthaceae	Ipopo ale, ajikewu	103335	Leaf, Stem	Herb
87	*Grewia pubescens* P.Beauv.	Malvaceae	Oraigbo	103336	Leaf	Shrub
88	*Harungana madagascariensis* Lam. ex Poir.	Hypericaceae	Amuje	103337	Leaf, Stem	Tree
89	*Heliotropium indicum* L.	Boraginaceae	Ogbe akuko	103338	Leaf	Herb
90	*Hibiscus rosa-sinensis* L.	Malvaceae	Isapa	103339	Leaf	Shrub
91	*Hibiscus surattensis* L.	Malvaceae	Ewe – emu, *Isapa pupa*	103340	Stem, Root	Climber
92	*Hippocratea pallens* Planch. ex Oliv. (Synonym: *Apodostigma pallens* (Planch. ex Oliv.) R.Wilczek)	Celastraceae	Oju-ologbo-nla	103341	Leaf	Climber
93	*Hoslundia opposita* Vahl	Lamiaceae	Iwaregbo	103342	Leaf	Shrub
94	*Hyptis suaveolens* (L.) Poit.	Lamiaceae	Jogbo, Arunfonfon	103343	Leaf, Stem	Herb
95	*Icacina trichantha* Oliv.	Icacinaceae	Gbegbe	103344	Leaf, Stem	Shrub
96	*Indigofera arrecta* A.Rich.	Fabaceae	Elu-aja	103345	Leaf	Herb

97	*Indigofera hirsuta* L.	Fabaceae	Ejaọmode	103346	Leaf, Stem	Herb
98	*Ipomoea involucrata* P.Beauv. (Synonym: *Ipomoea pileata* Roxb.)	Convolvulaceae	Ododo odo, Elukeresi	103347	Leaf, Stem, Root	Herb
99	*Jatropha curcas* L.	Euphorbiaceae	Botuje, Lapalapa	103348	Leaf	Shrub/Small tree
100	*Jatropha gossypiifolia* L.	Euphorbiaceae	Lapalapa pupa	103349	Leaf, Root	Shrub
101	*Kigelia africana* (Lam.) Benth.	Bignoniaceae	Pandoro	103350	Stem-bark, Root	Tree
102	*Lantana camara* L.	Verbenaceae	Ewon-agogo, efinrin oso	103351	Leaf, Stem	Shrub
103	*Lawsonia inermis* L.	Lythraceae	Laali	103352	Leaf, Stem	Shrub
104	*Leucaena leucocephala* (Lam.) de Wit	Fabaceae	Lusina	103353	Root	Tree
105	*Luffa cylindrica* (L.) M.Roem.	Cucurbitaceae	Kankan ayaba, Aya oyinbo	103354	Leaf, Stem	Climber
106	*Macaranga barteri* Müll.Arg.	Euphorbiaceae	Araasa, Awasa	103355	Leaf, Stem	Tree
107	*Microdesmis puberula* Hook.f. ex Planch.	Pandaceae	Ido Apata	103356	Leaf	Shrub
108	*Mallotus oppositifolius* (Geiseler) Müll.Arg.	Euphorbiaceae	Ipa, Eja, Oju-eja, Jeja, Iyadudu, Orokoro	103357	Stem, Root	Shrub/Tree
109	*Malvastrum coromandelianum* (L.) Garcke	Malvaceae	Asa, Olowonransansan, Sekuseku, Asa orisa, Aborisawaye	103358	Stem	Subshrub
110	*Mangifera indica* L.	Anacardiaceae	Mongoro	103359	Leaf, Stem-bark	Tree

111	*Margaritaria discoidea* (Baill.) G.L.Webster	Phyllanthaceae	Agaga-odan, Awe	103360	Leaf	Tree
112	*Mariscus alternifolius* Vahl (Synonym: *Cyperus cyperoides* (L.) Kuntze)	Cyperaceae	Alubosa eranko, Ikeregun, Samikoko, Efo'aba	103361	Leaf, Root	Sedge
113	*Melanthera scandens* (Schumach. & Thonn.) Roberty	Asteraceae	Abo yúnyún, Agbugbo	103362	Leaf, Stem	Climber
114	*Merremia pterygocaulos* (Choisy) Hallier f.	Convolvulaceae	Atewegbore	103363	Leaf	Climber
115	*Mezoneuron benthamianum* Baill. (Synonym: *Caesalpinia benthamiana* (Baill.) Herend. & Zarucchi)	Fabaceae	Aseta, Jenifinrani, amuranju	103364	Leaf	Climbing shrub
116	*Mimosa pudica* L.	Fabaceae	Padimo, patanmo	103365	Leaf, Stem	Creeper
117	*Morinda lucida* Benth.	Rubiaceae	Oruwo	103366	Stem	Tree
118	*Moringa oleifera* Lam.	Moringaceae	Ewe igbale	103367	Stem	Tree
119	*Myrianthus arboreus* P.Beauv.	Urticaceae	Obisere, Eweade, Ibishere, Eweure	103368	Leaf	Shrub/Tree
120	*Nauclea latifolia* Sm. (Synonym: *Sarcocephalus latifolius* (Sm.) E.A.Bruce)	Rubiaceae	Egbesi	103369	Leaf	Tree
121	*Newbouldia laevis* (P.Beauv.) Seem.	Bignoniaceae	Akoko	103370	Leaf	Shrub/Tree
122	*Ocimum gratissimum* L.	Lamiaceae	Efinrin	103371	Leaf, Root	Herb
123	*Panicum scandens* (Schrad. ex Schult.) Trin. (Synonym: *Setaria scandens* Schrad.)	Poaceae	Eeran esin	103372	Leaf	Herb
124	*Passiflora foetida* L.	Passifloraceae	Nine-nine	103373	Leaf, Stem	Creeping vine
125	*Paullinia pinnata* L.	Sapindaceae	Kakansela	103374	Leaf	Climber
126	*Perotis indica* (L.) Kuntze	Poaceae	Perotis	103375	Leaf	Herb

127	*Persea americana* Mill.	Lauraceae	Pia, apoka	103376	Leaf	Tree
128	*Phaulopsis falcisepala* C.B.Clarke (Synonym: *Phaulopsis ciliata* (Willd.) Hepper)	Acanthaceae	Atapara, Ata-igbó, Apa ọgbẹ́	103377	Leaf	Herb
129	*Phyllanthus amarus* Schumach. & Thonn.	Phyllanthaceae	Eyin olobe	103378	Leaf, Stem, Root	Herb
130	*Phyllanthus niruri* L.	Phyllanthaceae	Eyin olobe	103379	Leaf, Root	Herb
131	*Physalis angulata* L.	Solanaceae	Koropo	103380	Root	Herb
132	*Piliostigma thonningii* (Schum.) Milne-Redh. (Synonym: *Bauhinia thonningii* Schum.)	Fabaceae	Abafe	103381	Stem-bark	Tree
133	*Pinus caribaea* Morelet	Pinaceae	Pine	103382	Leaf	Tree
134	*Pleioceras barteri* Baill.	Apocynaceae	Dagba, Abeji, Abeko	103383	Leaf, Root	Climbing shrub, Liana
135	*Polyalthia suaveolens* Engl. & Diels (Synonym: *Greenwayodendron suaveolens* (Engl. & Diels) Verdc.)	Annonaceae	Agudugbu, Ooro	103384	Stem	Tree
136	*Portulaca oleracea* L.	Portulacaceae	Semolapa	103385	Leaf	Herb
137	*Psidium guajava* L.	Myrtaceae	Girofa	103386	Leaf	Tree
138	*Rauvolfia vomitoria* Afzel.	Apocynaceae	Asofeyeje	103387	Leaf, Root	Tree
139	*Ricinus communis* L.	Euphorbiaceae	Ewe –laa	103388	Leaf	Shrub
140	*Scoparia dulcis* L.	Plantaginaceae	Olorunyinni, eso ojuologbo Ominsinminsin Gogoro	103389	Leaf	Herb

141	*Secamone afzelii* (Roem. & Schult.) K.Schum.	Asclepiadaceae	Ailu, Arilu, Òlógbọn gbũrú	103390	Leaf	Shrub
142	*Securinega virosa* (Roxb. ex Willd.) Baill. (Synonym: *Flueggea virosa* (Roxb. ex Willd.) Royle)	Phyllanthaceae	Iranje, Awewe	103391	Stem, Root	Shrub
143	*Senna hirsuta* (L.) H.S.Irwin & Barneby	Fabaceae	Rere, Arunfofo	103392	Leaf, Root	Shrub
144	*Senna obtusifolia* (L.) H.S.Irwin & Barneby	Fabaceae	Ako-rere, Epa ikun	103393	Leaf	Herb
145	*Senna podocarpa* (Guill. & Perrottet) Lock	Fabaceae	Asuwon ibile, Aja-rere	103394	Leaf	Shrub
146	*Senna siamea* (Lamarck) H.S.Irwin & Barneby	Fabaceae	Ayagba	103395	Root, Stem-bark	Tree
147	*Senna tora* (L.) Roxb.	Fabaceae	Epa-ikun, Ewe morin, Epaja-abo, *Aidan toro*	103396	Stem, Root	Herb
148	*Sesamum indicum* L.	Pedaliaceae	Ekuku gogoro	103397	Root	Herb
149	*Sesamum radiatum* Schumach. & Thonn.	Pedaliaceae	Ekuku Gogoro	103398	Root	Herb
150	*Sida acuta* Burm.f.	Malvaceae	Osekutu, isepotu, gbegi	103399	Leaf	Shrub
151	*Sida cordifolia* L.	Malvaceae	Iso-obo, Isekutu	103400	Leaf, Stem	Undershrub
152	*Sida linifolia* Juss. ex Cav.	Malvaceae	Osekutu	103401	Stem	Subshrub
153	*Smilax kraussiana* Meisn. (Synonym: *Smilax anceps* Willd.)	Smilacaceae	Ekanamagbo	103402	Root	Climbing shrub
154	*Solanum nigrum* L. (Synonym: *Solanum americanum* Mill.)	Solanaceae	Igba, odu	103403	Stem	Herb
155	*Solanum torvum* Sw.	Solanaceae	Ewe Igbalode, Igbalode legun	103404	Root	Shrub/ Small tree

156	*Solenostemon monostachyus* (P.Beauv.) Briq. (Synonym: *Plectranthus monostachyus* (P.Beauv.) B.J.Pollard)	Lamiaceae	Olojongbodu, Aranpolo	103405	Leaf, Stem	Herb
157	*Sorghum bicolor* (L.) Moench	Poaceae	Okababa	103406	Stem	Herb
158	*Sphenocentrum jollyanum* Pierre	Menispermaceae	Akerejupoo	103407	Seed, Root	Shrub
159	*Spigelia anthelmia* L.	Loganiaceae	Aparan	103408	Leaf, Stem	Herb
160	*Spondias mombin* L.	Anacardiaceae	Iyeye	103409	Leaf, Stem, Root	Tree
161	*Sporobolus indicus* (L.) R.Br.	Poaceae	Motisan	103410	Leaf, Stem	Herb
162	*Stachytarpheta indica* (L.) Vahl	Verbenaceae	Iru eku, Iru alangba, Akisan	103411	Leaf, Root	Shrub
163	*Sterculia tragacantha* Lindl.	Malvaceae	Alawefon, Okagbo	103412	Root	Tree
164	*Synedrella nodiflora* (L.) Gaertn.	Asteraceae	Ako-yun-yun, Aworo-ona	103413	Leaf, Root	Herb
165	*Syzygium guineense* (Willd.) DC.	Myrtaceae	Adere, Igi aro	103414	Leaf	Tree
166	*Talinum triangulare* (Jacq.) Willd. (Synonym: *Talinum fruticosum* (L.) Juss.)	Talinaceae	Gure	103415	Leaf, Root	Herb
167	*Tapinanthus globiferus* (A.Rich.) Tiegh.	Loranthaceae	Afomo	103416	Root	Shrub
168	*Telfairia occidentalis* Hook.f.	Cucurbitaceae	Eweroko, Ugwu	103417	Leaf	Climber
169	*Terminalia ivorensis* A.Chev.	Combretaceae	Afara	103418	Leaf	Tree
170	*Tetracera alnifolia* Willd.	Dilleniaceae	Opon	103419	Leaf	Climber
171	*Thaumatococcus daniellii* (Benn.) Benth.	Marantaceae	Ewe iran	103420	Leaf	Herb

172	*Thevetia peruviana* (Pers.) K.Schum.	Apocynaceae	Kanminko	103421	Leaf, Root	Tree
173	*Tithonia diversifolia* (Hemsl.) A.Gray	Asteraceae	Agbale, Sepeleba	103422	Root	Shrub
174	*Trichilia monadelpha* (Thonn.) J.J.de Wilde	Meliaceae	Ajanrere, Ako rere	103423	Leaf	Tree
175	*Tridax procumbens* (L.) L.	Asteraceae	Igbalode, sábárúmá	103424	Leaf, Root	Herb
176	*Triumfetta cordifolia* A.Rich.	Malvaceae	Yogboro, Akee-eri	103425	Leaf, Stem	Shrub
177	*Urena lobata* L. var. *glauca* (Blume) Borss. Waalk.	Malvaceae	Okeriri	103426	Leaf	Shrub
178	*Urena lobata* L.	Malvaceae	Oju oro, Okeriri, Ilasa agborin, Ilasa omode, Ilasa oyibo	103427	Leaf, Stem	Shrub
179	*Vernonia amygdalina* Delile	Asteraceae	Ewuro	103428	Leaf, Stem	Shrub
180	*Vernonia cinerea* (L.) Less. (Synonym: *Cyanthillium cinereum* (L.) H.Rob.	Asteraceae	Amunimuye, Bojure	103429	Leaf, Stem, Root	Herb
181	*Vitex doniana* Sweet	Lamiaceae	Oori	103430	Root	Tree
182	*Waltheria indica* L.	Malvaceae	Korikodi, Ewe epo	103431	Leaf	Shrub
183	*Zingiber officinale* Roscoe	Zingiberaceae	Atale	103432	Root	Herb

Table S2. Folkloric uses of the identified plants and the number of participants from each location. LGA = local government area

S/N	Plant	Imota (Epe LGA)	Ijebu (Epe LGA)	Alimosho LGA	Badagry LGA	Eti-Osa LGA
		Health conditions/diseases treated				
1	*Abrus precatorius*	Stomach ache (15)	Cough (18)	Nill	Nill	Cough (20)
2	*Acalypha fimbriata*	Nill	Nill	Cough (12)	Divinity (19)	Nill
3	*Acanthospermum hispidum*	Nill	Hypertension (17)	Nill	Divinity(10)	Hypertension (18)
4	*Achyranthes aspera*	Erection (20)	Sight (15)	Potency (17)	Nill	Sight (19)
5	*Adenia lobata*	Pile (16)	Arthritis (18)	Nill	Nill	Arthritis (14)
6	*Ageratum conyzoides*	Infectious disease (17)	Malaria (20)	Hypertension (16)	Sight (13)	Malaria (14)
7	*Albizia ferruginea*	Cough (12)	Pregnancy (14)	Nill	Nill	Nill
8	*Albizia lebbeck*	Nill	Fever (12), Arthritis (8)	Arthritis (18)	Cough (19)	Nill
9	*Albizia zygia*	Aphrodisiac (7), Arthritis (13)	Cough (17), Toothache (3)	Cough (9), Arthritis (11)	Arthritis (17)	Nill
10	*Alchornea cordifolia*	Sight (12), Arthritis (8)	Ulcers (13), Arthritis (7)	Malaria (16), Sight (4)	Sight (12), Malaria (6)	Sight (5)
11	*Alchornea laxiflora*	De-worming (14), Infectious diseases (6)	Malaria (20)	Nill	Oral hygiene (17)	Nill
12	*Alternanthera sessilis*	Nill	Nill	Sight (11)	Jaundice (6)	Nill
13	*Amaranthus spinosus*	Nill	Nill	Sight (9)	Cirrhosis (12)	Nill
14	*Amaranthus viridis*	Nill	Nill	Longevity (13)	Nill	Nill
15	*Anacardium occidentale*	Nill	Malaria (20)	Nill	Nill	Malaria (18)
16	*Anchomanes difformis*	Chicken pox (8), measles (12)	Nill	Nill	Nill	Nill
17	*Annona senegalensis*	Malaria (16)	Nill	Nill	Potency (14)	Nill
18	*Anthocleista djalonensis*	Nill	Hypertension (15)	Nill	Nill	Hypertension (11)
19	*Anthocleista vogelii*	Nill	Nill	Nill	Divinity (7)	Nill
20	*Artocarpus communis* (Synonym: *Artocarpus altilis*)	Nill	Nill	Nill	Malaria (20)	Nill
21	*Aspilia africana*	Fibroid (13)	Cough (17)	Nill	Purgative (9)	Cough (11)
22	*Asystasia gangetica*	Neck pain (8)	Sight (13)	Potency (10)	Cough (18)	Sight (15)
23	*Azadirachta indica*	Nill	Nill	Nill	Anti-snake bite (16)	Nill
24	*Baphia nitida*	Divinity (12)	Nill	Nill	Nill	Nill

25	*Barleria opaca*	Nill	Nill	Diabetes (17)	Nill	Nill
26	*Bidens pilosa*	Nill	Nill	Cough (19)	Nill	Nill
27	*Boerhavia diffusa*	Nill	Nill	Malaria (18)	Nill	Nill
28	*Borreria scabra* (Synonym: *Spermacoce ruelliae)*	Ringworm (14)	Diabetes (16)	Eczema (16)	Birth control (17)	Diabetes (10)
29	*Borreria verticillata (Synonym: Spermacoce verticillata)*	Ringworm (18)	Ringworm (20)	Birth control (5)	Nill	Nill
30	*Bridelia ferruginea*	Diabetes (16)	Diabetes (19)	Nill	Birth control (18)	Nill
31	*Bryophyllum pinnatum*	Nill	Cough (20)	Sight (15)	Nill	Cough (19)
32	*Caladium bicolor*	Nill	Pimples (17)	Cough (18)	Nill	Pimples (12)
33	*Calophyllum inophyllum*	Nill	Nill	Nill	Scurvy (8)	Nill
34	*Calopogonium mucunoides*	Ulcer (14)	Scurvy (3), Cough (11)	Cough (19)	Nill	Scurvy (8), Cough (9)
35	*Calotropis procera*	Nill	Nill	Malaria (15)	Nill	Conjunctivitis (10)
36	*Canna indica*	Nill	Cirrhosis (11)	Pimples (14)	Nill	Cirrhosis (3)
37	*Canavalia ensiformis*	Nill	Pregnancy (4)	Nill	Nill	Pregnancy (6)
38	*Carpolobia lutea*	Easy delivery (12)	Nill	Nill	Nill	Nill
39	*Celosia argentea*	Nill	Nill	Malaria (2)	Nill	Nill
40	*Centrosema pubescens*	Nill	Nill	Pimples (6)	Purgative (7)	Nill
41	*Chassalia kolly*	Nill	Nill	Aphrodisiac (12)	Aphrodisiac (6)	Nill
42	*Chromolaena odorata*	Malaria (20)	Nill	Potency (4), Malaria (18)	Nill	Malaria (20)
43	*Cissampelos owariensis*	Miscarriage (20)	Nill	Nill	Miscarriage (16)	Nill
44	*Citrus aurantiifolia*	Nill	Malaria (19)	Nill	Nill	Malaria (7)
45	*Cleistopholis patens*	Nill	Pimples (13)	Nill	Nill	Pimples (10)
46	*Cleome fruticosa* (*Cadaba fruticosa*)	Nill	Nill	Cirrhosis (13)	Birth control (8)	Nill
47	*Clerodendrum capitatum*	Malaria (14)	Nill	Nill	Diabetes (12)	Nill
48	*Clerodendrum paniculatum*	Nill	Nill	Cirrhosis (6)	Nill	Nill
49	*Clerodendrum umbellatum*	Nill	Nill	Stomach ache (16)	Nill	Nill
50	*Clerodendrum volubile*	Nill	Nill	Nill	Immune booster (2)	Nill

51	*Cnestis ferruginea*	Nill	Immune booster (4)	Nill	Nill	Immune booster (1)
52	*Cola millenii*	Miscarriage (9)	Nill	Nill	Nill	Nill
53	*Colocasia esculenta*	Nill	Nill	Cough (14)	Nill	Nill
54	*Commelina africana*	Curse (3)	Nill	Nill	Diabetes (11)	Nill
55	*Commelina erecta*	Nill	Nill	Potency (15)	Nill	Nill
56	*Costus afer*	Ulcer (16)	Cough (19) Ulcer (1)	Cough (18)	Ulcer (5)	Ulcer (10) Cough (8)
57	*Croton lobatus (Astraea lobata)*	Nill	Nill	Nill	Cough (18)	Nill
58	*Croton zambesicus* (*Croton gratissimus*)	Nill	Nill	Hypertension (10)	Malaria (13)	Nill
59	*Cucumeropsis* mannii	Nill	Nill	Cough (1)	Nill	Nill
60	*Cyathula prostrata*	Nill	Nill	Nill	Pimples (3)	Nill
61	*Cymbopogon citratus*	Nill	Nill	Longevity (9)	Nill	Nill
62	*Cyperus haspans*	Nill	Nill	Arthritis (11)	Nill	Nill
63	*Dalbergia saxatilis*	Longevity (14)	Nill	Nill	Nill	Nill
64	*Datura metel*	Hypertension (14)	Nill	Immune booster (11)	Muscle relaxant (4)	Nill
65	*Desmodium velutinum*	Erection (1)	Nill	Nill	Nill	Nill
66	*Dichrostachys cinerea*	Headache (10)	Toothache (12)	Nill	Cough (15)	Nill
67	*Dissotis rotundifolia (Heterotis rotundifolia*)	Easy delivery (6)	Pregnancy care (11)	Nill	Nill	Pregnancy care (12)
68	*Eclipta prostrata* (*Heterotis rotundifolia)*	Nill	Nill	Cough (16)	Nill	Nill
69	*Elaeis guineensis*	Nill	Nill	Nill	Blood tonic (3)	Nill
70	*Eleusine indica*	Pile (9)	Cough (7)	Nill	Malaria (17)	Cough (13)
71	*Eleutheranthera ruderalis*	Nill	Nill	Pile (16)	Longevity (10)	Nill
72	*Emilia coccinea*	Nill	Foot pains (14)	Birth control (3)	Purgative (12)	Foot pains (8)
73	*Entandrophragma angolense*	Pile (15)	Nill	Nill	Nill	Nill
74	*Eragrostis namaquennsis* (*Eragrostis japonica*)	Nill	Stomach ache (7)	Nill	Nill	Stomach ache (3)
75	*Erigeron floribundus*	Nill	Nill	Scurvy (9)	Nill	Nill

76	*Erythrina senegalensis*	Nill	Nill	Nill	Diabetes (13)	Nill
77	*Euphorbia glaucophylla* (*Euphorbia trinervia*)	Nill	Longevity (17)	Malaria (11)	Cough (15)	Longevity (6)
78	*Euphorbia heterophylla*	Nill	Nill	Longevity (1)	Nill	Nill
79	*Ficus benjamina*	Nill	Scurvy (5), Cough (14)	Scurvy (1), Cough (7)	Nill	Scurvy (4), Cough (2)
80	*Ficus capensis* (*Ficus sur*)	Nill	Hypertension (7), Cough (18)	Nill	Nill	Hypertension (5), Cough (15)
81	*Ficus exasperata*	Scurvy (9), Cough (12)	Hypertension (10)	Scurvy (8)	Scurvy (5)	Nill
82	*Ficus polita*	Nill	Nill	Nill	Pimples (6)	Nill
83	*Fleurya aestuans*	Nill	Nill	Cough (18)	Nill	Nill
84	*Gliricidia sepium*	Nill	Nill	Arthritis (12)	Hypertension (14)	Nill
85	*Glyphaea brevis*	Ulcer (13)	Nill	Nill	Nill	Nill
86	*Gomphrena celosioides*	Nill	Pimples (10)	Nill	Potency (14)	Pimples (13)
87	*Grewia pubescens*	Nill	Nill	Nill	Hypertension (16)	Nill
88	*Harungana madagascariensis*	Anti-snake bite (11)	Anti-snake bite (6)	Nill	Nill	Anti-snake bite (1)
89	*Heliotropium indicum*	Erection (9)	Nill	Nill	Pile (18)	Nill
90	*Hibiscus rosa-sinensis*	Nill	Nill	Cough (10)	Nill	Nill
91	*Hibiscus surattensis*	Nill	Diabetes (15)	Nill	Potency (12)	Diabetes (8)
92	*Hippocratea pallens* (*Apodostigma pallens*)	Malaria (17)	Nill	Nill	Nill	Nill
93	*Hoslundia opposita*	Ulcer (19)	Nill	Nill	Nill	Nill
94	*Hyptis suaveolens*	Nill	Anti-snake bite (15)	Nill	Nill	Anti-snake bite (7)
95	*Icacina trichantha*	Curse (14)	Potency (15)	Potency (10)	Potency (17)	Potency (11)
96	*Indigofera arrecta*	Nill	Nill	Pile (11)	Pregnancy (7)	Nill
97	*Indigofera hirsuta*	Sight (9)	Stomach ache (16)	Sight (5)	Purgative (19)	Stomach ache (12)
98	*Ipomoea involucrata* (*Ipomoea pileata*)	Nill	Anti-snake bite (13), Cough (17)	Malaria (16)	Cough (20)	Anti-snake bite (3), Cough (12)
99	*Jatropha curcas*	Pile (12)	Nill	Pile (19)	Sight (9)	Pile (15)
100	*Jatropha gossypiifolia*	Nill	Piles (20)	Nill	Nill	Piles (20)
101	*Kigelia africana*	Cough (13)	Nill	Nill	Pile (15)	Nill

102	*Lantana camara*	Nill	Nill	Purgative (7), Nervousness (1)	Potency (6)	Nill
103	*Lawsonia inermis*	Nill	Malaria (18)	Gonorrhoea (10)	Nill	Malaria (16)
104	*Leucaena leucocephala*	Nill	Nill	Purgative (6)	Nill	Nill
105	*Luffa cylindrica*	Cramps (7)	Fever (15)	Purgative (19)	Convulsion(9)	Nill
106	*Macaranga barteri*	Easy delivery (14)	Anti-snake bite (15)	Nill	Nill	Anti-snake bite (4)
107	*Microdesmis puberula*	Easy delivery (11)	Nill	Nill	Nill	Nill
108	*Mallotus oppositifolius*	Stomach problem (16)	Nill	Malaria (10)	Sight (5)	Nill
109	*Malvastrum coromandelianum*	Nill	Nill	Anti-snake bite (2)	Nill	Nill
110	*Mangifera indica*	Malaria (19)	Malaria (16)	Malaria (19)	Malaria (11) Fever (8)	Fever (14) Malaria (8)
111	*Margaritaria discoidea*	Nill	Malaria (13)	Nill	Nill	Malaria (10)
112	*Mariscus alternifolius* (*Cyperus cyperoides*)	Nill	Nill	Jaundice (3)	Arthritis (6)	Nill
113	*Melanthera scandens*	Curse (9)	Nill	Nill	Malaria (15)	Nill
114	*Merremia pterygocaulos*	Nill	Foot pain (11)	Sight (4)	Nill	Foot pain (5)
115	*Mezoneuron benthamianum* (*Caesalpinia benthamiana*)	Piles (11)	Nill	Nill	Pile (14)	Nill
116	*Mimosa pudica*	Nill	Erection (14)	Anti-snake bite (4)	Nill	Erection (7)
117	*Morinda lucida*	Malaria (13)	Fever (17)	Jaundice (9)	Malaria (17)	Malaria (14)
118	*Moringa oleifera*	Nill	Cough (17)	Nill	Nill	Cough (10)
119	*Myrianthus arboreus*	Neck pain (12)	Cough (5)	Nill	Nill	Nill
120	*Nauclea latifolia* (*Sarcocephalus latifolius*)	Malaria (16)	Malaria (17), Pimples (10)	Nill	Diabetes (15)	Pimples (8)
121	*Newbouldia laevis*	Diabetes (15)	Measles (17)	Worm-expellant (7)	Jaundice (16)	Diabetes (12)
122	*Ocimum gratissimum*	Piles (18)	Purgative (20)	Purgative (16)	Cough (12)	Piles (12)

123	*Panicum scandens* (*Setaria scandens*)	Nill	Malaria (13)	Nill	Nill	Malaria (8),
124	*Passiflora foetida*	Nill	Foot pain (16)	Nill	Sight (6)	Foot pain (9)
125	*Paullinia pinnata*	Pile (11)	Aphrodisiac (12)	Nill	Nill	Nill
126	*Perotis indica*	Nill	Nill	Blood tonic (3)	Nill	Nill
127	*Persea americana*	Nill	Nill	Nill	Longevity (14)	Nill
128	*Phaulopsis falcisepala* (*Phaulopsis ciliata*)	Malaria (15)	Nill	Nill	Nill	Nill
129	*Phyllanthus amarus*	Fever (11)	Pregnancy (13)	Immune booster (8)	Cough (11)	Pregnancy (4)
130	*Phyllanthus niruri*	Nill	Foot pain (6)	Nill	Sight (8)	Foot pain (5)
131	*Physalis angulata*	Skin rashes (20)	Skin rashes (18)	Nill	Pile (12)	Nill
132	*Piliostigma thonningii* (*Bauhinia thonningii*)	Nill	Nill	Potency (6)	Nill	Nill
133	*Pinus caribaea*	Nill	Nill	Nill	Cough (5)	Nill
134	*Pleioceras barteri*	Neck pain (7)	Nill	Stomach ache (11)	Nill	Nill
135	*Polyalthia suaveolens* (*Greenwayodendron suaveolens*)	Nill	Nill	Cough (2)	Nill	Nill
136	*Portulaca oleracea*	Nill	Nill	Cough (10)	Anti-snake bite (8)	Nill
137	*Psidium guajava*	Fever (9)	Stomach ache (17)	Nill	Nill	Stomach ache (14)
138	*Rauvolfia vomitoria*	Malaria (10), Insanity (18)	Hypertension (14)	Muscle relaxant (7)	Cough (10)	Muscle relaxant (3)
139	*Ricinus communis*	Nill	Nill	Divinity (13)	Sight (16)	Nill
140	*Scoparia dulcis*	Black coated tongue (4)	Nill	Nill	Birth control (9)	Nill
141	*Secamone afzelii*	Nill	Immune booster (14)	Nill	Nill	Immune booster (12)
142	*Securinega virosa* (*Flueggea virosa*)	Typhoid (10)	Typhoid (12)	Pregnancy (13)	Immune booster (11)	Nill
143	*Senna hirsuta*	Nill	Nill	Cough (10)	Purgative (17)	Nill
144	*Senna obtusifolia*	Nill	Scurvy (9), Cough (13)	Nill	Nill	Scurvy (7), Cough (12)
145	*Senna podocarpa*	Stomach Problem (16)	Nill	Nill	Nill	
146	*Senna siamea*	Nill	Nill	Nill	Immune booster (11)	Nill

147	*Senna tora*	Nill	Potency (11), Sight (5)	Malaria (13)	Divinity (4)	Potency (1), Sight (2)
148	*Sesamum indicum*	Nill	Nill	Nill	Potency (2)	Potency (1)
149	*Sesamum radiatum*	Nill	Potency (6)	Nill	Nill	Nill
150	*Sida acuta* Burm.	Fibroid (7)	Sight (6)	Sight (3)	Nill	Sight (2)
151	*Sida cordifolia*	Nill	Cough (8)	Nill	Nill	Cough (4)
152	*Sida linifolia*	Nill	Scurvy (9)	Nill	Pimples (7)	Scurvy (1)
153	*Smilax kraussiana* (*Smilax anceps*)	Erection (12)	Nill	Nill	Nill	Nill
154	*Solanum nigrum* (*Solanum americanum*)	Nill	Nill	Diabetes (15)	Cough (17)	Nill
155	*Solanum torvum*	Pimples (8)	Potency (10)	Hypertension (13)	Nill	Potency (5)
156	*Solenostemon monostachyus* (*Plectranthus monostachyus*)	Pile (14)	Malaria (10)	Purgative (9)	Potency (11)	Malaria (8)
157	*Sorghum bicolor*	Nill	Nill	Malaria (7)	Nill	Nill
158	*Sphenocentrum jollyanum*	Deworming (8)	Malaria (6)	Deworming (6)	Fever (11)	Nill
159	*Spigellia anthelmia*	Nill	Nill	Diabetes (10)	Pile (16)	Nill
160	*Spondias mombin*	Penile erection (14)	Diabetes (8)	Anti-snake bite (5)	Insomnia (6)	Diabetes (2)
161	*Sporobolus indicus*	Nill	Diabetes (10)	Purgative (9)	Nill	Diabetes (4)
162	*Stachytarpheta indica*	Erection (4)	Hypertension (9)	Nill	Nill	Hypertension (7)
163	*Sterculia tragacantha*	Diabetes (13)	Nill	Nill	Nill	Nill
164	*Synedrella nodiflora*	Potency (10) Sight (1)	Nill	Longevity (5)	Malaria (2)	Nill
165	*Syzygium guineense*	Nill	Nill	Diabetes (12)	Nill	Nill
166	*Talinum triangulare* (*Talinum fruticosum*)	Blood tonic (20)	Blood tonic (18)	Malaria (6)	Nill	Nill
167	*Tapinanthus globiferous*	Fibroid (11)	Nill	Nill	Nill	Nill
168	*Telfairia occidentalis*	Blood tonic (19)	Blood tonic (16)	Hypertension (9)	Nill	Nill
169	*Terminalia ivorensis*	Divinity (9)	Nill	Nill	Nill	Nill
170	*Tetracera alnifolia*	Fibroid (5)	Nill	Nill	Nill	Nill
171	*Thaumatococcus daniellii*	Ulcer (16)	Ulcer (13)	Food sweetener (15)	Ulcer (17)	Food sweetener (1), Ulcer (3)

172	*Thevetia peruviana*	Purgative (14)	Purgative (10)	Pimples (7)	Cough (8)	Nill
173	*Tithonia diversifolia*	Malaria (18)	Malaria (15)	Malaria (11)	Pile (9)	Malaria (12)
174	*Trichilia monadelpha*	Insomnia (2)	Nill	Nill	Nill	Nill
175	*Tridax procumbens*	Ulcer (8)	Foot pains (9)	Malaria (7)	Hypertension (9)	Foot pains (3)
176	*Triumfetta cordifolia*	Easy delivery (9)	Nill	Birth control (5)	Nill	Nill
177	*Urena lobata* var. *glauca*	Nill	Potency (10)	Nill	Nill	Potency (5)
178	*Urena lobata*	Birth control (11)	Rheumatism (10)	Wound (17)	Diarrhea (13)	Stomach ache (8)
179	*Vernonia amygdalina*	Diabetes (15)	Malaria (13)	Oral hygiene (17)	Diarrhea (16)	Malaria (18)
180	*Vernonia cinerea* (*Cyanthillium cinereum*)	Asthma (14)	Bronchitis (11)	Cold (8)	Stomachache (11)	Cold (5)
181	*Vitex doniana*	Dysentery (11)	Diarrhea (14)	Dysentery (10)	Hypertension (14)	Indigestion (9)
182	*Waltheria indica*	Wound (13), Blood tonic (19)	Ulcer (16), Blood tonic (12)	Cold (10)	Cough (18)	Cough (5)
183	*Zingiber officinale*	Ulcer (12), Indigestion (14)	Cough (14), Ulcer (9)	Indigestion (10)	Cough (18)	Flu (10)

Table S3. Data collection sheet for ethnobotanical survey of medicinal plants used for managing various health conditions/diseases in five (5) selected locations in Lagos State of Nigeria

	**Plant**	**Disease**	**Part used** (Leaves=0), (Stem=1), (Bark =2), (Seeds=5), (Roots=3), (Flower=4) (Whole plant =6) (Rhizome/bulb=7)	**Preparation** (Maceration = 0); Poultice=1; Decoction 2. Enema=3; infusion=4	**Plant form** (shrub, herbs, trees, climber)	**Administration** (Topical=0), (Orally=1), (Bathing=2), (Enema=3)	**Dosage** (0=1x/day), (1=1-3x/day), (2=as needed and 3= other	**Abundance** (Common=0), (Rare=1), (Abundant=2)
1								
2								
3								
4								
5								
6								
7								
8								
9								
10								
11								
12								
13								
14								
15								
